# Sirtuins and cognition: implications for learning and memory in neurological disorders

**DOI:** 10.3389/fphys.2022.908689

**Published:** 2022-07-22

**Authors:** Eric Fagerli, Iris Escobar, Fernando J. Ferrier, Charles W. Jackson, Efrain J. Perez-Lao, Miguel A. Perez-Pinzon

**Affiliations:** Department of Neurology, Miller School of Medicine, University of Miami, Miami, FL, United States

**Keywords:** sirtuins, cognition, memory, ischemia, Alzheimer’s disease, oxidative stress, synaptic plasticity, epigenetics

## Abstract

Sirtuins are an evolutionarily conserved family of regulatory proteins that function in an NAD^+^ -dependent manner. The mammalian family of sirtuins is composed of seven histone deacetylase and ADP-ribosyltransferase proteins (SIRT1-SIRT7) that are found throughout the different cellular compartments of the cell. Sirtuins in the brain have received considerable attention in cognition due to their role in a plethora of metabolic and age-related diseases and their ability to induce neuroprotection. More recently, sirtuins have been shown to play a role in normal physiological cognitive function, and aberrant sirtuin function is seen in pathological cellular states. Sirtuins are believed to play a role in cognition through enhancing synaptic plasticity, influencing epigenetic regulation, and playing key roles in molecular pathways involved with oxidative stress affecting mitochondrial function. This review aims to discuss recent advances in the understanding of the role of mammalian sirtuins in cognitive function and the therapeutic potential of targeting sirtuins to ameliorate cognitive deficits in neurological disorders.

## 1 Introduction

Cognitive function, including memory, attention, decision making, perception, and language comprehension, is important in daily life at any age (Frith, 1981). Due to the umbrella coverage of cognition over many areas, proper cognitive function serves a crucial role in basic behaviors and social interactions. Cognitive function declines with age and causes significant impairments in the quality of life in millions of patients. While cognitive function declines with age, neurological disorders can also contribute to cognitive deterioration.

Sirtuins have generated significant attention since the discovery that silent information regulator 2 (Sir2) proteins not only acted as genetic silencing factors in *Saccharomyces cerevisiae* (Klar et al., 1979; Rine et al., 1979) but also were found to modulate lifespan (Kaeberlein et al., 1999; Kim et al., 1999). Sirtuins are an evolutionarily conserved family of NAD^+^-dependent class III histone deacetylases (HDACs) due to their ability to remove acetyl groups from lysine residues on histone proteins; however, they are also known to act on nonhistone proteins as well as to remove additional acyl groups from lysine residues (Frye, 1999; Shirakawa et al., 2013). Thus, it has become apparent that sirtuins act as energy sensors as well as transcriptional effectors due to the NAD^+^-dependent regulation of the acetylation state of histones and other transcriptional regulators.

**FIGURE 1:**
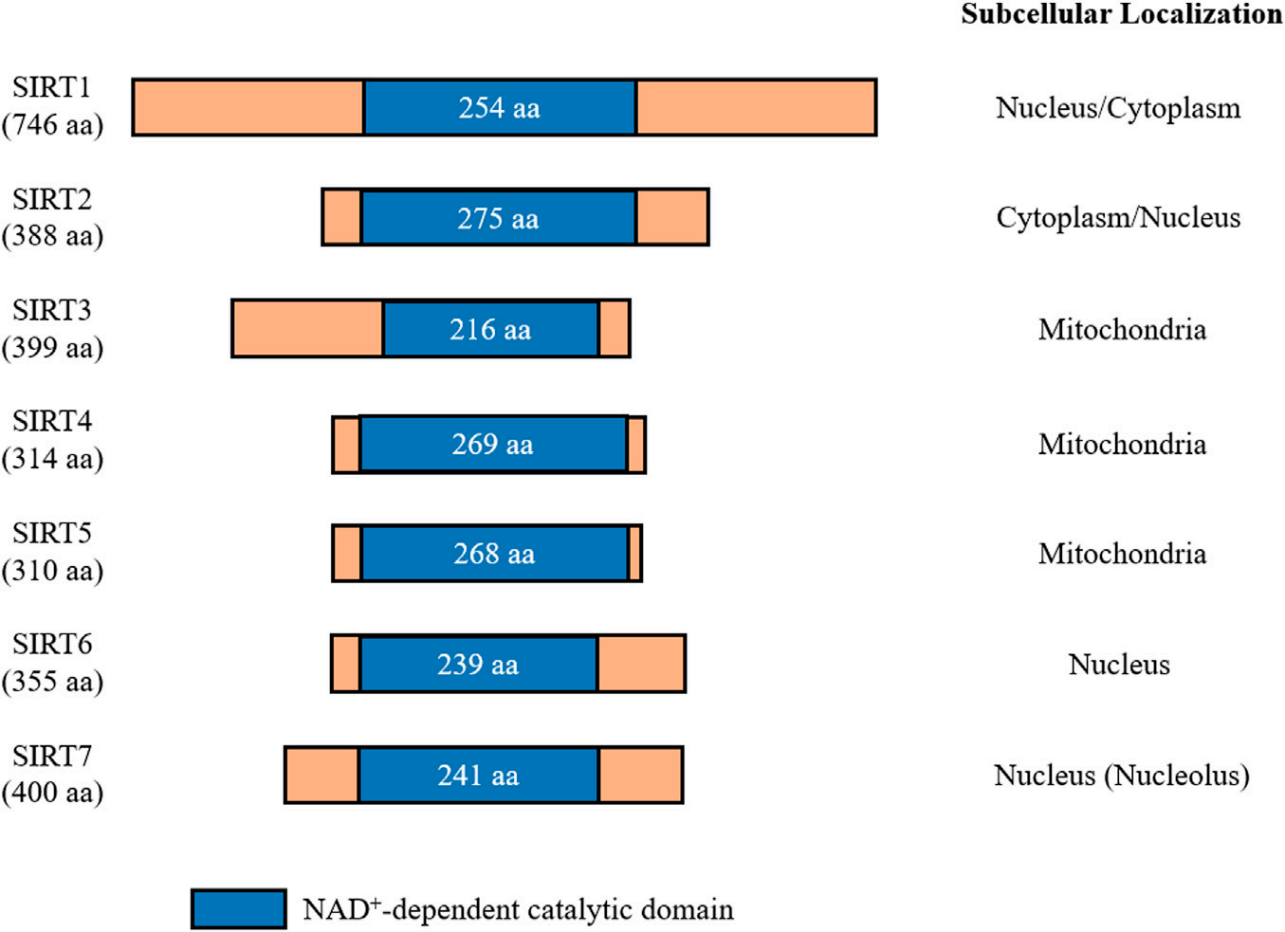
Mammalian sirtuins share a conserved catalytic core domain comprising between 200 and 275 amino acids. This catalytic domain requires NAD^+^ to be present for catalytic function. Each member of the sirtuin family is classified by their localization within cells. Amino acids (aa).

In mammals, the sirtuin family consists of seven proteins (SIRT1-7), which all share an evolutionarily conserved catalytic domain of 200-275 amino acids with variable N- and C-terminal domains (Canto et al., 2015) (Figure 1). These different sirtuin proteins vary in subcellular localization, tissue specificity, enzymatic activity, and targets for interaction. SIRT1 is mainly localized in the nucleus, but it is also present in lower levels in the cytosol due to nuclear export shuttling under specific conditions, such as when the insulin pathway is inhibited or oxidative stress is induced (Tanno et al., 2007; Yanagisawa et al., 2018). SIRT2 is a cytosolic protein that is present in the nucleus during the G2 to M phase transition of the cell cycle, and it is suggested to play a role in chromatin condensation (Vaquero et al., 2006). SIRT3, SIRT4, and SIRT5 are mitochondrial sirtuins with mitochondrial targeting sequences (Haigis et al., 2006; Schwer et al., 2006; Hallows et al., 2008; Nakagawa et al., 2009). While both SIRT6 and SIRT7 are nuclear sirtuins, SIRT7 is reported to reside in the nucleolus (Liszt et al., 2005; Kiran et al., 2013) (Figure 1).

In this review, we present our current understanding of the sirtuin family concerning contribution to the brain and cognitive function. We focus on the role sirtuins play in both physiological and pathophysiological conditions to elucidate the importance of targeting sirtuins as a therapeutic avenue to relieve cognitive deficits from neurological conditions, primarily cerebral ischemia (CI) and Alzheimer’s disease (AD).

## 2 Understanding cognitive deficits after cerebral ischemia in clinical cases

While the brain only constitutes about 2% of total body weight, it consumes around 20% of total body energy (Clarke and Sokoloff, 1999). Due to the high energy requirements of the brain, it is highly susceptible to ischemic injuries such as cardiac arrest (CA) and ischemic or hemorrhagic stroke (Clarke and Sokoloff, 1999; Sims and Muyderman, 2010; Rundek and Sacco, 2015). Even with decades of studies and research, CI remains a leading cause of death and disability worldwide (Avan and Hachinski, 2021). Following CI, surviving patients may develop dysfunction to one or more cognitive domains, with executive function, attention, and memory being the domains most commonly affected (Carlson et al., 2009; Moulaert et al., 2009; Perez et al., 2016). Recombinant tissue plasminogen activator, which is a thrombolytic agent, remains the only FDA-approved treatment for ischemic stroke; however, it is administered to less than 5% of stroke patients due to an administrative window within 3 h, or 4.5 h in certain eligible cases, of the onset of clotting symptoms (Lansberg et al., 2009; Jilani and Siddiqui, 2021). No true prophylactics are currently available to prevent CI or post-CI cognitive decline (van der Velden, 1988). Thus, new avenues of research are needed to focus on pursuing novel drug targets to combat CI.

The assessment of post-CI cognitive impairment is typically based on neuropsychological evaluations, which are limited in terms of accuracy and objectivity. Additionally, they are prone to the influence of age and education. Therefore, the selection of which neuropsychological test to use is important. For example, the Cerebral Performance Category Scale and the Modified Rankin Scale are designed to test gross neurological function and overall functional disability respectively, but they are not able to distinguish between patients with differing degrees of cognitive impairments. Thus, other assessments have been designed and employed to assess the various degrees of cognitive impairments following CI. The Mini-Mental State Examination (MMSE) was developed to ascertain a patient’s cognitive state, but it can only detect more moderate to severe cognitive deficits in patients (Folstein et al., 1975; Jokinen et al., 2015). The Montreal Cognitive Assessment (MCoA) is designed to be more sensitive than the MMSE. Therefore, both the MMSE and MCoA are commonly used in assessing cognition post-CI (Godefroy et al., 2011; Chiti and Pantoni, 2014; Shen et al., 2016; van Gils et al., 2022).

### 2.1 Clinical evidence of cognitive impairments following ischemic stroke

Cognitive impairments are common among patients after ischemic stroke. Following a patient’s first ischemic stroke, the prevalence of dementia within the first-year ranges from 9 to 30% in hospital-based studies (Srikanth et al., 2004). However, the prevalence of post-stroke cognitive impairment with no dementia is likely much higher. In a study that recruited patients with different subtypes of stroke, MMSE performance was significantly worse compared to the non-stroke control group 6 months following the stroke event (Kase et al., 1998). Another study utilizing a battery of neuropsychological tests showed that 3 months following a stroke, 83% of patients showed impairment in one or more cognitive realms, and 50% of patients were impaired in 3 or more domains (Jokinen et al., 2015).

Cognitive recovery after stroke may occur spontaneously following stroke. It may take weeks, months, or even years if it occurs at all (Cramer, 2018). In cases where there is post-stroke cognitive impairment with no dementia, only 10% of the stroke patients recovered cognitive function within 1 year (Rasquin et al., 2005). However, clinical studies suggest that the yearly rate of developing dementia following a stroke is about twice as high as the general population and has a rate that is 3.83 times higher when compared to healthy controls (Kokmen et al., 1996; Desmond et al., 2002).

Among surviving stroke patients, the risk of stroke recurrence is reported to range from 7.0 to 20.6% over the first year (Kokmen et al., 1996; Salgado et al., 1996; Kase et al., 1998; Desmond et al., 2002; Srikanth et al., 2004; Rasquin et al., 2005; Xu et al., 2007; Mohan et al., 2011; Cramer, 2018) and from 16.2 to 35.3% over the first 5 years (Hata et al., 2005; Mohan et al., 2009; Mohan et al., 2011). Having recurrent strokes greatly increases the risk of dementia, with an estimated incidence of over 40% in patients that experience recurrent strokes (Pendlebury and Rothwell, 2009). Thus, the short- and long-term impacts of ischemic stroke plague a significant portion of surviving stroke patients.

### 2.2 Clinical evidence of cognitive impairments following cardiac arrest

CA is characterized by a cessation of blood flow resulting from a sudden stoppage of the heart from beating. CA is also associated with cognitive deficits, and the prevalence of cognitive impairments in survivors of CA is nearly 50% (Perez et al., 2016). Longer episodes of cardiac arrest lead to greater risks of cognitive impairment. Common impairments after CA include dysfunction of short-term memory, attention, immediate and delayed recall, and executive function (Medrzycka-Dabrowska et al., 2018).

Moderate or severe memory impairments between in-hospital and out-of-hospital patients differed when utilizing the Rivermead Behavioral Memory Test for episodic memory. Cognitive deficits were found in 26% of in-hospital and 38% of out-of-hospital patients 8 months following CA (O'Reilly et al., 2003). A recent study showed that compared to patients that experienced a myocardial infarction, CA patients have 6 times the likelihood of developing cognitive impairments (Byron-Alhassan et al., 2021). The incidence of dementia in surviving patients is also significantly higher after sudden CA according to the MMSE (Jaszke-Psonka et al., 2016).

In a longitudinal study that followed patients for 4 years following CA, 29% of patients displayed cognitive impairments on the Cambridge Neuropsychological Test Automated Battery (Buanes et al., 2015). This battery of tests is a computer assessment that indicated damage to the medial temporal lobe, which is utilized in short-term memory and executive function (Buanes et al., 2015). Additional computerized assessment utilizing the Computerized Assessment for Mild Cognitive injury also found cognitive deficits in CA patients (Sabedra et al., 2015). Computerized testing for cognitive impairments following CI may provide a more detailed assessment of cognitive impairments that could improve testing in a fast, sensitive, and more reliable manner. Computer assessments may prove to be more consistent and more cost-effective in long-term cognitive studies following CI.

## 3 Understanding cognitive deficits in dementia

Dementia is characterized by progressive deterioration in two or more cognitive domains, thus causing lower quality of life through increased difficulty of basic daily function. AD is the most common cause of dementia worldwide, accounting for up to 80% of all dementia cases (Crous-Bou et al., 2017). The prevalence of AD is continuing to grow as the population ages. Due to the increasing prevalence of AD, direct and indirect healthcare costs are estimated upwards of $500 billion annually (Takizawa et al., 2015). While AD plays a massive role in disrupting normal life due to cognitive decline, this review will focus on the contributions of CI to AD and vascular cognitive impairment (VCI). For a thorough discussion of AD studies on cognitive impairment, the readers are guided to the following reviews, among others, that further elaborate on the contributions of AD in cognitive impairment (Corey-Bloom, 2002; Lyketsos et al., 2011; Kirova et al., 2015; Takizawa et al., 2015; Bondi et al., 2017; Crous-Bou et al., 2017; Kivipelto et al., 2018; Weller and Budson, 2018; Cummings et al., 2019; Knopman et al., 2021).

Following a CI event such as stroke or CA, disruption of blood flow can persist in surviving patients due to damaged and weakened cerebrovasculature, resulting in subsequent hypoperfusion. VCI may be used to describe any cognitive impairments that are associated with cerebrovascular disease, and hypoperfusion has been identified as one of the main culprits of VCI. VCI ranges from mild cognitive impairments to vascular dementia that is caused by ischemic or hemorrhagic stroke and vascular factors alone or in a combination with neurodegeneration such as that from AD (Roman, 2002; Rundek et al., 2021). Cerebral hypoperfusion will lead to blood-barrier disruption, microglial activation, matrix metalloproteinase activation, white matter lesions, and ultimately, cognitive impairment due to chronic glucose and oxygen deficiency in parenchymal tissue (Swartz et al., 2003; Kitamura et al., 2012; Liu and Zhang, 2012; Zietemann et al., 2016; Duncombe et al., 2017; Zilberter and Zilberter, 2017). Clinically, VCI is best diagnosed and characterized by identifying the presence and quantifying the extent of progressive cognitive or functional deficits and the identification of vascular brain injury through neuroimaging (Seshadri et al., 2015).

### 3.1 Contribution of cerebral ischemia to dementia

Stroke has been shown to significantly increase the risk of developing dementia. Studies suggest stroke doubles the risk for dementia (Pendlebury and Rothwell, 2009), and approximately 30% of stroke patients develop cognitive impairments within 3 years of the event (Savva et al., 2010). There is an estimated 1.59 times increased risk for AD following stroke (Zhou et al., 2015), and the increased risk is 1.5 times the general population risk in the first year alone (Kokmen et al., 1996). With regard to AD, clinical evidence suggests that following stroke, there are pathological changes that have AD-like pathology. Abnormal amyloid-β (Aβ) deposition and hyperphosphorylated tau are prominent features of AD. Increased Aβ deposition is clinically linked to patients that experience ischemic stroke (Lee et al., 2005; Goulay et al., 2020). Cerebral Aβ deposition was determined to be significantly associated with post-stroke cognitive impairments in a 3-year longitudinal study that utilized the MMSE and MCoA annually following the ischemic event (Liu W. et al., 2015). Memory, visuospatial, and executive function domains were all significantly disrupted and had a significant relation to the level of Aβ deposition (Liu W. et al., 2015). Levels of total tau were found to increase in patient blood (De Vos et al., 2017) and cerebrospinal fluid samples after acute ischemic stroke (Hesse et al., 2001; De Vos et al., 2017). Detection of the presence and level of tau protein is negatively associated with the long-term outcome of stroke patients (Bielewicz et al., 2011; De Vos et al., 2017). Deposition of both proteins has been observed at higher levels in postmortem hippocampal tissue of stroke patients (Akinyemi et al., 2017).

Additionally, studies have shown that there was an average elevation in serum Aβ levels by 7-fold at 10 h after CA compared to control patients, and the magnitude of the increase correlates with decreased cognitive outcome when cognition was assessed 6 months later (Zetterberg et al., 2011). Postmortem hippocampal tissue shows increased deposition of Aβ plaques in patients that experienced CA compared to non-CA controls (Wisniewski and Maslinska, 1996; Qi et al., 2007). Increased cerebrospinal fluid tau and serum tau were observed in CA patients as well (Randall et al., 2013; Rosen et al., 2014). A study that measured serum tau up to 72 h following CA found that the increased serum tau was associated with increased cognitive impairment 6 months following CA (Mattsson et al., 2017).

Findings in ischemic stroke patients are similar to the findings in CA patients concerning the development of AD-like pathology following the CI event. There are likely similar pathological mechanisms that occur resulting in neuronal loss, Aβ deposition, and tau hyperphosphorylation that occurs in these disease states, but further research is needed to provide evidence confirming this relationship.

## 4 Implications of sirtuins in cognition

All seven members of the sirtuin family are known to be expressed in the brain, and neurological conditions including CI and AD differentially regulate the expression and activation of different sirtuins. Given that SIRT1 is the best-studied member of the mammalian sirtuins, the majority of published literature on this topic focuses on SIRT1. However, in recent years, there has been increasing evidence through the use of animal models of neurological disorders that other sirtuins - namely SIRT2, SIRT3, and SIRT6 - also play a critical role in regulating cognitive function.

### 4.1 Sirtuin regulation of synaptic plasticity

Synaptic plasticity refers to structural and functional alterations within synapses that result in the strengthening or weakening of synaptic contacts. The synapse’s ability to undergo such changes is critical for information processing, learning, and memory encoding. Notably, studies have highlighted a role for sirtuins in modulating synaptic plasticity under basal conditions as well as pathological states.

Sirt1 was first implicated as an important mediator of synaptic plasticity from genetic deletion studies investigating long-term potentiation (LTP)—a form of long-term synaptic plasticity associated with an increase in synaptic strength/efficacy. LTP at the hippocampal CA3-CA1 synapse was significantly impaired in germline whole-body SIRT1 knockout (KO) mice carrying two null alleles of *Sirt1* (Michan et al., 2010) and in brain-specific conditional knockout mice lacking SIRT1 catalytic activity (Gao et al., 2010). Findings from the latter study also demonstrated that brain-specific SIRT1 loss-of-function decreased the expression of synaptic plasticity-related markers and spine density within the hippocampus (Gao et al., 2010). Further investigation revealed that synaptic deficits were mediated, in part, by post-transcriptional regulation of cAMP response binding protein (CREB) *via* miR-134, which resulted in the translational block and downregulation of CREB protein expression (Gao et al., 2010). Recent evidence suggests SIRT2 and SIRT3 also function as key players for maintaining synaptic plasticity under basal conditions. SIRT2 has previously been shown to deacetylate AMPA receptors, in turn promoting their internalization and subsequent degradation *via* the ubiquitin-proteasome pathway (Wang G. et al., 2017). Interestingly, inhibition of SIRT2 increases cell-surface expression of AMPARs, and SIRT2 deficient mice exhibit impairments in hippocampal LTP and long-term depression (LTD), which indicates SIRT2-mediated regulation of AMPARs is necessary for synaptic plasticity (Wang G. et al., 2017). Under inflammatory conditions, microglial-specific SIRT2 deficient mice also exhibit LTP impairments (Sa de Almeida et al., 2020). Furthermore, a recent study showed that Sirt3 deletion significantly reduced LTP in the anterior cingulate cortex and the deficit was attributed to neuronal cell loss in this region (Kim et al., 2019).

Numerous studies have identified alterations in the expression/activity of sirtuins in several neurological disorders characterized by synaptic dysfunction (Julien et al., 2009; Hernandez-Jimenez et al., 2013; Kumar et al., 2013; Han et al., 2014; Kalaivani et al., 2014; Lutz et al., 2014; Erburu et al., 2015; Shimizu et al., 2016; Yin et al., 2018; Zhang Z. et al., 2019; Wang et al., 2019). While the extent to which alterations in sirtuin expression contribute to synaptic deficits has not been fully deduced under pathological conditions, studies utilizing pharmacological or genetic approaches to manipulate sirtuin activity/expression suggest they play key roles in either driving or attenuating synaptic pathology in disease states. In the case of AD, pretreatment with resveratrol - an activator of SIRT1 - was shown to reverse Aβ_1–42_-induced impairment of LTP (Wang R. et al., 2017). In an *App* ^*NL-G-F*^ mouse model of AD, an intermittent fasting diet rescued LTP deficits in the hippocampus, which was dependent on SIRT3 expression (Liu Y. et al., 2019). The same study also demonstrated that knockdown of Sirt3 in *App* ^*NL-G-F*^ mice significantly reduced hippocampal CA1 pyramidal neuron spine density (Liu Y. et al., 2019). Furthermore, increased expression of Sirt3 and/or Sirt6 induced by treatment with nicotinamide riboside or an analog 3-iodothyronamine was associated with improved LTP in different AD mouse models (Hou et al., 2018; Bellusci et al., 2020).

In terms of neuropsychiatric disorders, both SIRT1 and SIRT2 have been implicated in the epigenetic regulation of synaptic-plasticity related genes associated with depression and chronic stress; however, their exact roles remain controversial. While studies have indicated that SIRT1 induces positive effects on depressive-like behaviors as well as general synaptic function and ultrastructure in chronic unpredictable stress models (Abe-Higuchi et al., 2016; Shen et al., 2019; Lei et al., 2020), inhibition of SIRT1 expression reversed decreases in spine density within the ventral hippocampal CA1 region in a model of post-traumatic stress disorder (Li et al., 2019). Likewise, previous studies found that downregulation of SIRT2 induces anti-depressant-like effects and is associated with enhanced expression of synaptic plasticity-related markers (Erburu et al., 2017; Munoz-Cobo et al., 2018). In contrast, a separate study reported that reductions in SIRT2 expression induce depressive-like behaviors and concomitant downregulation of synaptic plasticity-related genes in mice (Wang et al., 2019). Aside from psychiatric disorders, SIRT1 has also been shown to mediate synaptic ultrastructural changes in addiction (Xia et al., 2018), alleviate neuropathic pain by modulating structural synaptic plasticity and LTP in spinal dorsal horn neurons (Zhang Z. et al., 2019; Zhong et al., 2019; Mei et al., 2020), and rescue aberrant changes in spine density induced by ischemia/reperfusion injury (Jokinen et al., 2015) or propofol exposure in neonatal rats (Ma et al., 2022). SIRT2 and SIRT3 have also been implicated in regulating synaptic plasticity following ischemia (Shimizu et al., 2016) as well as in rodent models of scopolamine- and anesthesia-induced memory impairment (Liu et al., 2021; Lu et al., 2021). It is clear that sirtuins play key roles in modulating different synaptic plasticity processes including synaptic remodeling, signaling, and neurotransmission. As such, sirtuins are emerging as promising targets to preserve synaptic function in various neurological diseases.

### 4.2 Sirtuin regulation of gene expression

Gene expression is the biological process of translating coded instructions from the genome into functional molecules that influence specific cell processes. Epigenetic modifications may affect gene function and expression and may also facilitate crosstalk between genes and the environment. SIRT1-7 have broad action roles in the epigenetic regulation in the cell. They have the potential to change gene expression and pathways utilizing a mechanism of lysine deacetylation, by which they are able to modify histones or nonhistone proteins to alter structure and functionality (Smith and Boeke, 1997). Among the sirtuin family, SIRT1, SIRT2, SIRT6, and SIRT7 are the most related to the process of chromatin remodeling and interaction with transcriptional related factors (Gupta et al., 2022). SIRT3-5 localize in mitochondria, and they target the mitochondrial genetic material or proteins related to cell metabolism and energy response (Gupta et al., 2022).

The epigenetic regulation of a transcriptional pathway could occur by direct or indirect interactions with histones. SIRT1 regulates genetic expression by deacetylating histone H3 in lysine 9 and 14 (H3K9ac, H3K14) and H4 in lysine 16 (H4K16ac), thus allowing the chromatin to open for transcription (Imai et al., 2000; Suka et al., 2002; Corpas et al., 2017). Overexpression of SIRT1 in the 3xTg-SIRT1 mice model for AD showed decreased symptoms of impairments in learning and memory, thus preserving cognition (Corpas et al., 2017). One possibility of this is that SIRT1 regulates protein expression in a similar strategy as it does in models of Huntington’s disease using transgenic BSKO-R6/2 mice. Expression of SIRT1 promoted BDNF expression by targeting the promoter region and consequently enhanced the CREB-TORC1 pathway activity (Jeong et al., 2011). However, another suggested mechanism for this model is related to the modification of the R3 and R7 regions upstream of the transcription frame for miRNA-134 (Gao et al., 2010). miRNA-134 is a transcriptional regulator of CREB and BDNF, and it is related to protecting cognition in 3xTg-SIRT1 mice (Wang G. et al., 2017). Another regulatory function of SIRT1 involves the direct interaction with other regulatory proteins. High concentrations of SIRT1 in serum are correlated to better cognition in Parkinson’s disease (PD) patients (Chen et al., 2021; Zhu et al., 2021). It has been described that SIRT1 deacetylates peroxisome proliferator-activated receptor γ coactivator-1α (PGC-1α), a key regulator of mitochondrial biogenesis and potential target for therapy in PD patients (Nemoto et al., 2005; Zheng et al., 2010; Wang et al., 2015). SIRT3 has also been shown to have a role in this particular pathway and is related to the quality control system of mitochondria during degenerative diseases (Meng et al., 2019). SIRT3 is reported to promote mitochondrial biogenesis by promoting PGC-1α expression (Fu et al., 2012).

There is evidence of SIRT2 regulation of genes related to memory and plasticity as well. Known targets of SIRT2 include histone H3 lysine 18 and 56 (H3K18ac) and histone H4K16ac (Vaquero et al., 2006; Das et al., 2009). Previous models associate SIRT2 with cognition, memory, and synaptic plasticity by the stabilization of AMPA receptors (Wang G. et al., 2017). Corepressor of RE1 silencing transcription (CoREST) factor may recruit SIRT2 to deacetylate H4K16ac to induce downregulation of GluA1 and GluA2 AMPA receptor subunits, thus decreasing synaptic plasticity and potentially cognition in a methamphetamine addiction model (Cadet and Jayanthi, 2013). A model with downregulation of SIRT2 in stress stimulus showed increased transcription of *Ehtm2* and accompanying reduced expression of crucial synaptic plasticity genes that encode proteins including Egr1, Synaptophysin, and Synapsin1 (Wang et al., 2019). Furthermore, inhibition of EHMT1/2 in the late-stage familial Alzheimer’s disease (FAD) mouse model has shown positive results in the recovery from cognitive impairment and synaptic functionality (Zheng et al., 2019). Therefore, there is the possibility that SIRT2 may have an important role as an epigenetic regulator of these genes in neurodegenerative diseases (Zheng et al., 2019).

SIRT6 also plays an important role in epigenetic regulation. In addition to its deacetylation enzymatic activity, SIRT6 also conveys increased ADP-ribosyltransferase activity compared to SIRT1 and SIRT2. SIRT6 has been associated with the regulation of age-related pathologies (Liszt et al., 2005). The main targets for SIRT6 deacetylation are histone 2B lysine 12 (H2BK12ac) and histone H3 lysine 9 and 56 (H3K9ac) (Michishita et al., 2008; Yang et al., 2009). Novel targets relating to the prevention of errors during mitosis and cell senescence have been identified in H3K18ac at the pericentric chromatin (Tasselli et al., 2016). In a SIRT6 conditional KO model in mice, impairment in contextual fear conditioning has been observed. This suggests a possible role of SIRT6 in the formation of contextual memory (Kim et al., 2018). In the 5xFAD transgenic mouse model, memory impairment and psychoemotional changes worsen with age (Grinan-Ferre et al., 2016). This model shows that SIRT6 is the only sirtuin that is downregulated at a relatively early age, and it may be related to global acetylation of histone H3 as well as other epigenetic abnormalities found in the AD brain (Kanfi et al., 2012; Grinan-Ferre et al., 2016). This suggests that SIRT6 is an important epigenetic regulator in the early degeneration and progression of the pathology (Kanfi et al., 2012). The role sirtuins play in epigenetic regulation is evident in a wide array of pathologies, and further understanding the role they play in gene modulation is an important avenue for therapeutic development in the amelioration of cognition in various neurological disorders.

### 4.3 Sirtuins and cognitive behavior in rodent animal models

Studies in mice have demonstrated that SIRT1 is highly implicated in modulating cognitive behavior. Mice with global SIRT1 overexpression displayed elevated anxiety, decreased exploratory drive, and increased susceptibility to depression by deacetylating the brain-specific transcription factor NHLH2, which increased its activity on the *MAO-A* promoter and therefore increased degradation of serotonin (Nordquist and Oreland, 2010; Libert et al., 2011). Furthermore, it was revealed that hippocampal SIRT1 activity increases in response to chronic variable stress, a rodent model of depression (Ferland and Schrader, 2011). However, it was unclear if the SIRT1 upregulation contributes to transcriptional dysregulation or acts as a positive-feedback mechanism to compensate for the stress effect (Ferland and Schrader, 2011).

Experiments in rats have shown that reducing excessive autophagy in a hippocampal SIRT1-dependent mechanism ameliorated cognitive impairments induced by sleep deprivation (Gao et al., 2021). SIRT1 has been shown to modulate the transcription of TFEB genes to promote autophagy (Huang et al., 2019). Autophagy is an essential homeostatic process in the clearance of protein aggregates involved in neurodegenerative diseases such as AD (Lashuel et al., 2002; Martini-Stoica et al., 2016). Thus, considering the role of SIRT1 in autophagy regulation, it is reasonable to suggest SIRT1 dysfunction is a contributing factor to autophagy dysfunction in these diseases. This is supported by a study suggesting that, following physical exercise (PE), initiation of SIRT1 signaling decreases Aβ production (Koo et al., 2017).

### 4.4 Sirtuins and physical exercise

Several studies have shown that SIRT1 and SIRT3 levels are regulated by PE. It was recently demonstrated that the known benefits of PE in cognitive functions and depressive symptoms involve lactate-dependent increases in BDNF expression when SIRT1 is activated (El Hayek et al., 2019). Moreover, PE-regulated SIRT1 activity also offers neuroprotective effects against the aging brain by suppressing hippocampal apoptosis and inflammation (Lin et al., 2020), which are known to be major causes of amnesia and dementia. SIRT1 activation by PE also modulates the transcription of TFEB genes to regulate autophagy (Huang et al., 2019). Furthermore, the benefits of SIRT1 caused by PE are not limited to the brain. Increased levels of SIRT1 induced by PE also occurred in heart cells to facilitate the adaptation of cardiac metabolism (Chen W. K. et al., 2018).

SIRT3 is regulated by other types of stimuli such as dieting and exercise, and it has been demonstrated that exercise produces an increase in the expression of SIRT3 (Liu Y. et al., 2019). PE through aerobic interval training has been shown to attenuate high-fat-diet-associated cognitive dysfunction through SIRT3 upregulation (Shi et al., 2018). In another model related to intermittent fasting (IF), SIRT3 activity was a key factor in the mechanism of synaptic adaptation of GABAergic synaptic transmission in hippocampus neurons (Cheng et al., 2016; Liu Y. et al., 2019). Sirt3(−/−) mice suffered low adaptability to IF, causing increased anxiety and poor memory retention (Cheng et al., 2016; Liu Y. et al., 2019). Knockout of SIRT6 using CRISP-Cas9 in cynomolgus monkeys shows SIRT6’s direct impact in neuronal differentiation, causing retardation in the development of neurons and the development of smaller brain sizes due to non-coding RNA H19 (Zhang et al., 2018).

Studies in elderly participants revealed that resistance PE training increases serum levels of SIRT1, SIRT3, SIRT5, and SIRT6 proteins after 12-weeks interventions (Hooshmand-Moghadam et al., 2020; Wasserfurth et al., 2021). Similarly, increased levels of SIRT3 and SIRT4 have been reported in many studies involving rats that underwent short-term (Lensu et al., 2019; Alavi et al., 2021; Heiat et al., 2021) and long-term PE (Nogueira-Ferreira et al., 2019). In sum, it seems clear that sirtuin regulation acts as one of the key mechanisms by which PE can improve cognitive functions, under both normal and pathological conditions.

## 5 Oxidative stress in cerebral ischemia and Alzheimer’s disease

As the sirtuin family is class III HDACs, they possess the ability to act as sensors of metabolism**.** The overproduction of reactive oxygen species (ROS) produces a condition known as oxidative stress (OS), where excessive ROS can damage cellular constituents and result in death (Sayre et al., 2008). Cell types that carry high metabolic demands, such as neurons, are more prone to OS (Pratico, 2008). In conditions such as CI and AD, OS is a major pathological contributor. Studies of postmortem AD brain tissue have shown increased ROS, reduced prevalence of antioxidants, and increased lipid peroxidation (Kopelovich Iu, 1975; Marcus et al., 1998; Beal, 2005; Garcia-Blanco et al., 2017; Ashraf et al., 2020). Hallmarks of AD such as mitochondrial dysfunction, metal accumulation, and build-up of neurofibrillary tangles and beta-amyloid plaques, heavily contribute to ROS production (Lovell et al., 1998; Pratico, 2008; Chen and Zhong, 2014; Cheignon et al., 2018). For instance, reactive metals in Aβ plaques enact the Fenton reaction to provide extracellular ROS while intracellular hyperphosphorylated tau can contribute to dysfunctional mitochondria, thus amplifying ROS levels (Sheard and Blair, 1970; Reddy and Oliver, 2019). Regarding CI, multiple cascading events contribute to an accumulation of ROS. Nutrient deprivation causes ATP depletion and compromises homeostasis. The majority of ROS comes from mitochondrial dysfunction, where reduced ATP and increased intracellular calcium impacts the mitochondrial membrane potential, dysregulates the electron transport chain, and reduces antioxidant capacity (Sheard and Blair, 1970; Pratico, 2008; Drose and Brandt, 2012; Sun et al., 2018). Additionally, ROS production can come from the activation of enzymes such as NADPH oxidases, which produce superoxides (Kahles and Brandes, 2012; Shenoda, 2015; Wu et al., 2018). OS is central to the pathologies of AD, CI, and many other neurological conditions. Neuroprotectants against OS are currently under investigation, and the family of sirtuins shows promise for such a role (Shahgaldi and Kahmini, 2021). In the following sections, we will detail the ability of sirtuins to protect against OS.

## 6 Role of sirtuins in oxidative stress

### 6.1 Cytosolic sirtuins

**SIRT2:** SIRT2 is highly expressed in the brain and its primary enzymatic activity is deacetylation, with mostly cytosolic targets. However, it has been linked to the deacetylation of certain histone residues (Khoury et al., 2018). SIRT2’s role in OS and neurodegeneration is a mixture of exacerbation and alleviation. SIRT2 expression was reported to increase in models that produce OS, such as H_2_O_2_ exposure, stroke, and normal aging (Pfister et al., 2008; Singh et al., 2017; Xie et al., 2017; Sarikhani et al., 2018). In HeLa cells, H_2_O_2_ exposure increased SIRT2 expression, inducing JNK deacetylation, phosphorylation, and increased activity. SIRT2 knockdown prevented these effects as well as reduced H_2_O_2_-induced cell death (Sarikhani et al., 2018). In ischemic models of middle cerebral artery occlusion (MCAO) *in vivo* and oxygen-glucose deprivation (OGD) *in vitro,* the experimental removal of SIRT2 was neuroprotective against both models. Additionally, the ischemic models increased SIRT2 levels (Xie et al., 2017). SIRT2 was increased in the cortex and hippocampus of experimentally aged rats and these rats also exhibited increased OS and the death-promoting factor FOXO3a (Keskin-Aktan et al., 2022). Enhanced SIRT2 activity can also be detrimental. In an SH-SY5Y model of PD, 6-OHDA treated cells showed increased SIRT2 phosphorylation and activity. GSK3β phosphorylated SIRT2 and in phosphorylation-resistant mutants, 6-OHDA toxicity was blocked (Liu S. et al., 2019). These studies indicate a relationship between OS and SIRT2. Moreover, they indicate SIRT2 plays a role in OS-mediated damage. These studies, however, are disputed by a collection of publications that indicate a positive role of SIRT2. SIRT2 has commonly been linked to the regulation of antioxidants. In SH-SY5Y cells, SIRT2 overexpression protected against diquat-induced OS through upregulation of superoxide dismutase (SOD) 2, while this effect was lost when SIRT2 was inhibited with AGK2 (Singh et al., 2017). SIRT2 increased nuclear NRF2 and Glutathione in PC12 cells treated with NADH, an effect lost with inhibition (Cao et al., 2016). In a similar study, NAD^+^ treatment-induced ERK activation and NRF2 expression, while SIRT2 siRNA blocked ERK activation (Zhang J. et al., 2019). SIRT2’s positive regulation of NRF2, and its resultant effect on antioxidants, shows a protective role against ROS directly. The protection of mitochondria also prevents ROS. Loss of SIRT2 in primary hippocampal neurons led to fragmented mitochondria and disrupted autophagy. In similar SIRT2-KO brain samples, ATP and antioxidants were depleted (Liu et al., 2017). With protective and deleterious evidence, the definitive role of SIRT2 in OS remains elusive.

### 6.2 Mitochondrial sirtuins

**SIRT3:** SIRT3 is the primary mitochondrial sirtuin, and its main enzymatic function is deacetylation. As a stress response enzyme, SIRT3 helps to regulate multiple mitochondrial functions, mostly through metabolism and ROS regulation. SIRT3 has been shown to deacetylate multiple protein targets of oxidative phosphorylation, the TCA cycle, the urea cycle, and mitochondrial DNA (mtDNA) stability (Gertz and Steegborn, 2016; Osborne et al., 2016; Khoury et al., 2018; Zheng et al., 2018; Zhang et al., 2020). Perhaps the most established target of SIRT3 is PGC-1α, which facilitates enhanced antioxidant enzyme expression and mitochondrial biogenesis (Shinohara et al., 1989; Shi et al., 2005; Kong et al., 2010; Ding et al., 2017). Specific targets of SIRT3 include the isocitrate dehydrogenase 2 (IDH2) enzyme to promote NADPH production, SOD2 to increase its antioxidant activity, and CypD to protect against the formation of the mitochondrial permeability transition pore (mPTP) (Yu et al., 2012; Zhang et al., 2016; Sun et al., 2017). Perturbations of SIRT3 have been associated with neurodegeneration and susceptibility to OS. In a 3-NPA mouse model of Huntington’s disease, SIRT3-KO mice showed reduced survival, increased striatal cell loss, and increased OS in primary neurons (Cheng et al., 2016). Postmortem brain tissue of AD patients showed reduced SIRT3, PGC-1a, and mitofusins 1 and 2, suggesting defective mitochondrial biogenesis and dynamics (Yin et al., 2020). ApoE4 AD mice showed significantly reduced SIRT3, PGC-1a, cognitive function, and ATP production. SIRT3 overexpression in primary neurons from these mice reversed these effects (Yin et al., 2019). In certain cases, SIRT3 is upregulated due to OS. H_2_O_2_ exposure *in vitro* has been shown to induce SIRT3. In such studies, further overexpression of SIRT3 provided neuroprotection through 1) prevention of mitochondrial Ca^2+^ overload 2) protection against intrinsic apoptosis and 3) maintenance of mitochondrial membrane potential through increased deacetylation of COX-1 (Dai et al., 2014; Tu et al., 2019). Similar to H_2_O_2_, OGD induces SIRT3 expression and further SIRT3 overexpression reduced H_2_O_2_ production, increased AMPK phosphorylation, and induced autophagy markers Beclin-1 and LC3-II (Yin et al., 2019). Studies *in vivo* also show increased SIRT3 as protective. In a model of intracerebral hemorrhage, the SIRT3 agonist Honokiol increased SIRT3 expression, promoted SOD deacetylation, and increased TFAM to protect mtDNA (Zheng et al., 2018). Finally, in an *in vivo* study of anesthesia/surgery-induced cognitive dysfunction in aged mice, SIRT3 overexpression in CA1 neurons protected against cognitive deficits while also promoting SOD and malondialdehyde antioxidant expression (Liu et al., 2021). SIRT3 appears to be upregulated or repressed in different OS contexts. Regardless, the overexpression of SIRT3 in OS environments produces neuroprotective outcomes.

**SIRT4:** SIRT4 shows lower homeostatic activity compared to the other mitochondrial sirtuins. SIRT4’s enzymatic activity is a combination of ADP ribosyl transferase, lipoamindase, and deacetylase functions (Osborne et al., 2016). SIRT4 appears to play an important role in the regulation of the NAD^+^/NADH ratio and NAD^+^ metabolism (Han Y. et al., 2019; Betsinger and Cristea, 2019). Additionally, SIRT4 has been connected to the regulation of gluconeogenesis (Khoury et al., 2018). Limited studies exist that highlight the potential role of SIRT4 in OS. SIRT4-KO mice exhibited exacerbated seizures and reduced GLT-1 expression in a kainic acid-induced seizure model. Additionally, ATP levels were reduced in primary hippocampal neurons of these mice (Shih et al., 2014). Shenfu Qiangxin Drink (SFQXD), a potential therapeutic, was shown to protect against H_2_O_2_ through reduced ROS levels, IL-1β, apoptosis, and increased FOXO3a phosphorylation. These effects were shown to be SIRT4-dependent (Zhang S. et al., 2021). Alternatively, some studies suggest SIRT4 increases susceptibility to disease. SIRT4 overexpression accelerated cardiac hypertrophy induced by Angiotensin II, through MnSOD inhibition (Luo et al., 2017; Betsinger and Cristea, 2019). SIRT4 may dysregulate mitochondrial dynamics by interacting with the protein OPA1 (Lang et al., 2017). Overall, SIRT4 appears to have a context-dependent influence on OS and OS-inducing conditions, presenting either protective or deleterious effects.

**SIRT5:** SIRT5 possesses multiple enzymatic activities including demalonylation, deglutarylation, desuccinylation, and deacetylation. In homeostasis, SIRT5 enacts regulation of metabolic processes such as glycolysis, fatty acid oxidation, and ROS detoxification (Khoury et al., 2018). Our laboratory has previously investigated SIRT5’s role in ischemic preconditioning and PKCε neuroprotection against MCAO in SIRT5-KO mice. These protective paradigms were determined to be SIRT5-dependent. SIRT5-KO mice showed increased succinylation of mitochondrial proteins, suggesting a supportive role of SIRT5’s mitochondrial desuccinylation functions (Morris-Blanco et al., 2016). Evidence suggests SIRT5 protects against ROS. In HEK292T cells, SIRT5 targeted SOD1 for desuccinylation, increasing its activity and reducing ROS levels (Lin et al., 2013). Cell lines deficient in SIRT5 displayed increased production of ROS following exposure to OS-inducing agents Sinopharm and paraquat. SIRT5’s succinylation of IDH2 and deglutarylation of G6PD promoted NADPH production and increased resilience to ROS (Zhou et al., 2016). Another desuccinylation target of SIRT5 is acyl-CoA oxidase 1 (ACOX1), which blocks ACOX1 dimerization and ROS production (Chen X. F. et al., 2018). Finally, SIRT5’s desuccinylation of pyruvate kinase M2 (PMK2) has been associated with increased resistance to ROS through multiple mechanisms (Hashtroudi et al., 1989; Wang F. et al., 2017; Qi et al., 2019). These studies indicate SIRT5’s capacity to protect against OS through its multiple enzymatic activities. SIRT5 may also be protective against OS-associated neurodegeneration. In APP/PS1 mice *in vivo*, and Aβ induced SH-SY5Y cells *in vitro*, SIRT5 overexpression protected against AD-induced OS, Aβ levels, and apoptosis. SIRT5 also induced SOD activity and reduced TNF-α and IL-6 signaling (Wu et al., 2021). SIRT5-KO mice in a subarachnoid hemorrhage (SAH) model highlighted desuccinylation targets of homeostatic metabolism. 211 lysine succinylation sites of 170 proteins, 39% of which were mitochondrial, were differential in these mice. Loss of SIRT5 exacerbated SAH, reduced ATP, and increased ROS (Xiao et al., 2021). Finally, a mouse model using MPTP-induced PD showed that SIRT5-KO mice exhibited increased motor deficits and ROS. SIRT5-KO exacerbated the MPTP-induced reduction in SOD of nigrostriatal dopaminergic neurons (Liu L. et al., 2015). SIRT5’s wide array of enzymatic activities and involvement in different OS contexts highlights it as a pivotal neuroprotective target.

### 6.3 Nuclear sirtuins

**SIRT1:** SIRT1 is the most heavily investigated member of the sirtuin family. SIRT1 predominantly enacts deacetylation function and has multiple enzyme and histone targets (Khoury et al., 2018). A myriad of neuroprotective effects of SIRT1 have been reported. One of SIRT1’s most detailed targets is the FOXO family of transcription factors. SIRT1 was shown to deacetylate these factors to induce the expression of antioxidants and to protect against OS-induced cell death (Brunet et al., 2004; Daitoku et al., 2004; van der Horst et al., 2004; Hori et al., 2013; Wang R. et al., 2017). Such antioxidants include SOD1, SOD2, catalase, HO-1, and more. SIRT1 can induce activation of NRF2 as well, promoting antioxidant expression (Ding et al., 2016; Shah et al., 2017; Han et al., 2018; Zhang X.S.et al., 2021). Targeting these transcription factors effectively deters OS. Some miRNAs, including miR-34, miRNA-23b-3p, miR132/212, and miR-134, target SIRT1 mRNA to limit the expression of the protein. This targeting of SIRT1 mRNA disrupts other pathways related to synaptic plasticity and the CREB, BDNF, Nrf2, and PTEN-PI3K pathways (Jeong et al., 2011; Wang et al., 2015; Han et al., 2018). To prevent ROS in the first place, SIRT1 promotes mitochondrial homeostasis and biogenesis through the deacetylation of PGC-1α, which in turn promotes expression of mitochondrial genes, protection of mtDNA, and metabolic function (Lagouge et al., 2006; Iwabu et al., 2010; Han B. et al., 2019). Inflammation is another process that can result in ROS overproduction and SIRT1 deacetylates the inflammatory factor NF-κB to deter inflammatory signaling. (Hwang et al., 2013; Bagul et al., 2015; Xu et al., 2019; Sun et al., 2021). A common result of NF-κB inhibition is a reduction in NADPH oxidase (NOX) isoforms, which mediate large amounts of superoxide in response to inflammatory signaling. Recent studies have highlighted SIRT1’s protection against OS in similar pathways. In a model of anesthetic neurotoxicity, sevoflurane administration induced suppression of SIRT1 expression and activated microglia in mouse pup hippocampi. Sevoflurane also induced IL-6 and TNF-α inflammatory signaling. Resveratrol-mediated SIRT1 activation prevented these effects and reduced NF-κB acetylation (Tang et al., 2021). SIRT1 was recently reported to be the target of neuroprotection against traumatic brain injury mediated by the natural compound astaxanthin. Oxidative stress, apoptosis, and neurological functions were all protected by the compound through SIRT1’s interaction with NRF2, however, SIRT1 inhibition with EX527 blocked these protective effects (Zhang X. S.et al., 2021). Calyxosin-7-O-beta-D-glucoside (CG), a promising protectant, protected against OGD in HT22 cells, a hippocampal line, through reduction of OS and apoptosis. This protection was associated with an increase in SIRT1, Foxo1, and PGC-1α (Yan et al., 2019). The wealth of new and old studies of SIRT1 highlight its pleiotropic nature for protection against OS across multiple neurodegenerative contexts.

**SIRT6:** SIRT6 possesses deacetylase, ADP ribosylase, and demyrisotylase activity. SIRT6 plays a major role in genomic stability through the protection of telomeres, facilitation of DNA damage repair, and cellular senescence (Khoury et al., 2018). Similar to SIRT2, the role of SIRT6 in neuronal injury has produced mixed results. SH-SY5Y cells overexpressing SIRT6 exhibited increased ROS production and necrotic cell death following OGD. SIRT6 inhibition provided protection against OGD through reduced autophagy (Shao et al., 2016). In primary mouse hippocampal neurons, SIRT6 overexpression exacerbated H_2_O_2_ induced neuronal death (Cardinale et al., 2015). These examples suggest exacerbation; however, most studies show SIRT6 plays a role to protect DNA from OS. SIRT6-deficient mice manifest genomic instability, while SIRT6-knockdown mouse embryonic fibroblasts present increased double-strand DNA breaks (Mao et al., 2011). Additionally, SIRT6 interacts with the Rad9-Rad1-Hus1 DNA repair enzymes, in an OS-dependent manner, to protect DNA telomeres from oxidative damage (Hwang et al., 2015). Additionally, SIRT6 has been associated with telomere mobility in response to oxidative damage (Gao Y. et al., 2018). SIRT6 activation is linked with NRF2 to protect against OS. Through repression of KEAP1, the NRF2 repressor protein, SIRT6 promotes NRF2 (Kanwal et al., 2019). Additionally, SIRT6 deacetylates H3K56 to help facilitate NRF2 binding to gene promoter regions (Pan et al., 2016). Finally, SIRT6 can bind to the NRF2 nuclear suppressor Bach1, which promotes detachment of Bach1 from NRF2 genomic targets (Ka et al., 2017; Yu et al., 2019). In both OGD, in N2a cells, and MCAO, in the mouse, SIRT6 overexpression protected against OS and ischemic injury through increased NRF2 levels (Zhang et al., 2017). In a D-gal and NaNO_2_ accelerated aging mouse model, the natural compound BaZiBuShen (BZBS) reversed aging-like OS through NRF2-induced increases in the antioxidant HO-1. This compound also increased mitochondrial oxygen consumption rate and Complex IV activity. Finally, BZBS preserved SIRT6 expression in the aging model (Li et al., 2022). There are some examples of SIRT6 playing a deleterious role in OS, however, most of the evidence suggests SIRT6 protects against OS through enhancing genomic stability and facilitating NRF2.

**SIRT7:** SIRT7 is the least investigated member of the sirtuin family. SIRT7 is localized to the nucleolus and exerts deacetylation and desuccinylase activity. A known histone target of SIRT7 deacetylation is H3K18ac and the reduction in this acetylation mark has been linked to tumor progression (Barber et al., 2012). SIRT7 has been linked to the positive regulation of ribosomal RNA transcription and regulation of RNA polymerase 1 *via* deacetylation. In addition to rRNA, SIRT7 has been linked with the regulation of tRNA transcription through indirect activation of RNA Polymerase III (Khoury et al., 2018). SIRT7’s role in OS in the CNS requires further investigation. Studies have suggested SIRT7 may promote tolerance to OS. SIRT7-KO mice exhibited perturbed oxidative phosphorylation, likely due to the loss of SIRT7’s regulation of NRF2 protein subunit GABPβ1 (Ryu et al., 2014). SIRT7-deficient mice presented a reduction in mean lifespans, increased heart hypertrophy, and increased inflammatory cardiomyopathy. Primary cardiomyocytes from these mice showed increased acetylation of p35 and increased susceptibility to OS (Vakhrusheva et al., 2008). Loss of SIRT7 was also shown to produce mitochondrial dysfunction and reduce ATP levels in primary mouse oocytes (Gao M. et al., 2018). Finally, SIRT7 desuccinylase activity at the H3K122 residue has been shown to promote chromatin condensation, which may protect against oxidative damage to the DNA in the event of OS (Li et al., 2016). Though evidence of SIRT7’s role in OS is limited, its involvement in mitochondrial function insinuates that there is more to be discovered.

## 7 Conclusion

**FIGURE 2:**
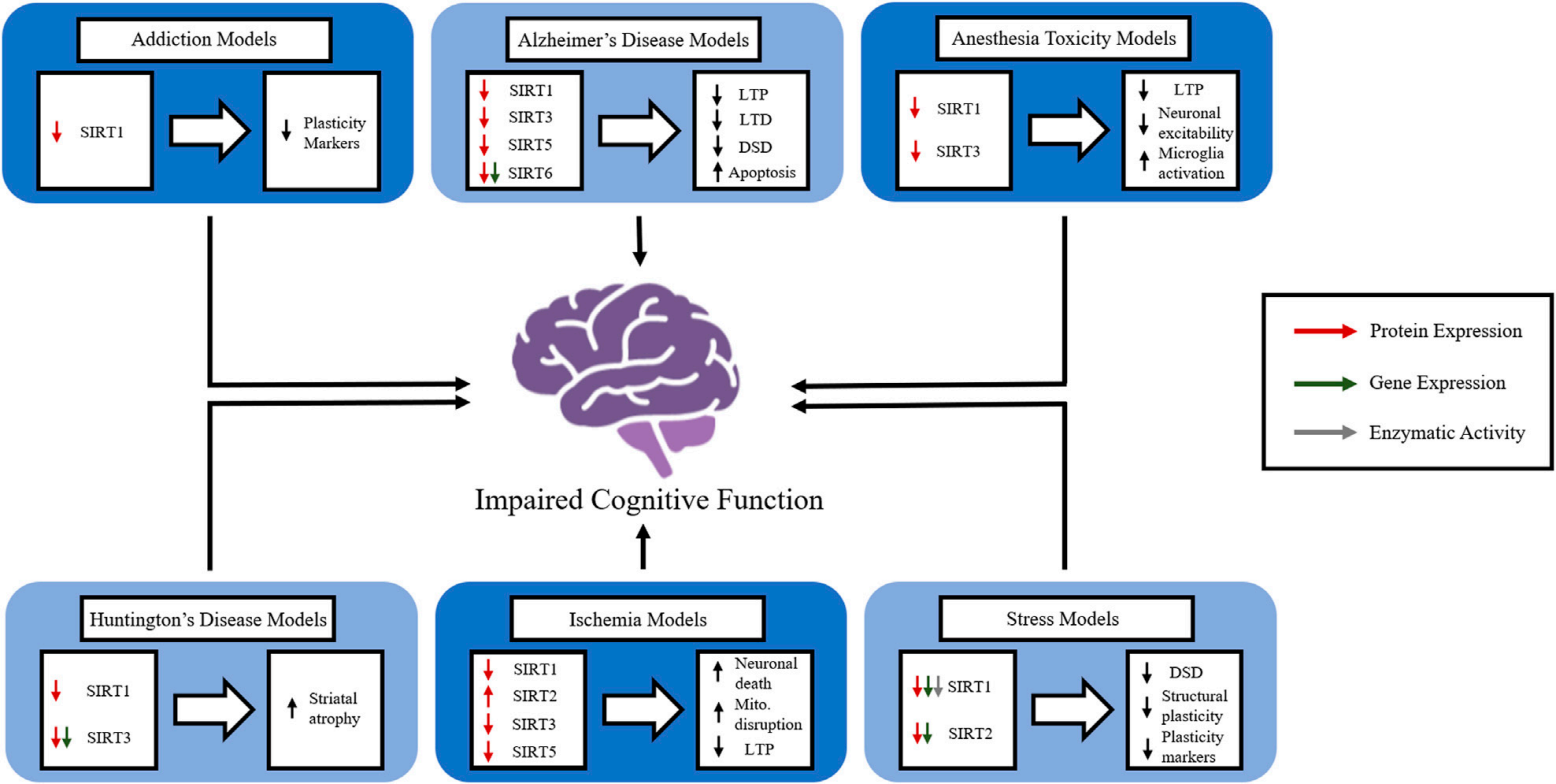
The role of members of the sirtuin family in *in vivo* models of various neurological disorders. The figure represents how individual sirtuins are affected in a variety of neurological disorders and how the disruption of sirtuin function causes disruptions in normal brain function. Long-term potentiation (LTP), long-term depression (LTD), dendritic spinal density (DSD), mitochondria (mito.).

Many neurological disorders have debilitating consequences and promote cognitive impairments that cause suffering and lower quality of life for those affected. The damage these disorders create is complicated and multifaceted, and further investigation is required to elucidate effective potential therapies. In spite of evidence supporting the importance of sirtuins in cognition, their precise functions and mechanisms of action in the brain remain largely unknown. The general activity and role of sirtuins in memory in the absence of disease states remain largely unknown, but their aberrant function in disease states suggests that they play a crucial role in physiological memory function. The contribution of sirtuins in the epigenetic regulation of synaptic protein expression and dendritic density offers insight into the role sirtuins play in enhancing the strength and/or efficacy of synaptic plasticity. When compounded with the role sirtuins play in molecular pathways involved with oxidative stress affecting mitochondrial function, the importance of targeting sirtuins as a therapeutic avenue for enhancing cognition becomes clear. However, further data on integrating the disruption of the many cellular physiological processes of sirtuins in neurological disorders is needed. Although we primarily focus on CI and AD in this review, it is likely that other neurological disorders cause or increase the risk of dementia through similar dysfunction of sirtuin activity (Figure 2). Thus, the more we understand the underlying mechanisms of sirtuins and their role in cognition in physiological and pathophysiological conditions, the more we will be able to manipulate and correct imbalances in cellular homeostasis to provide protection from and/or the amelioration of cognitive deficits.
